# Supplementary material for: Effect of honey and insulin treatment on oxidative stress and nerve conduction in an experimental model of diabetic neuropathy Wistar rats
Source: PLoS One. 2021 Jan 15;16(1):e0245395. doi: 10.1371/journal.pone.0245395 (PMC7810291; doi:10.1371/journal.pone.0245395)
Supplement: S1 File — (PDF) [file pone.0245395.s001.pdf]

Kejriwal
BEE CARE INDIA (PVT.) LTD.

Certificate of Analysis

Issue to: Kejriwal Bee Care India Pvt. Ltd

1. Product: Honey
2. Date of performance: 11-16.05.2019
3. B.No.: KBF-8347
4. Best Before: 18 Months from date of production
5. Date of Packing: 16.05.2019

TEST RESULTS

S. No.	Particulars	Result	As per The Food Safety and Standard Act, 2006 Specification	Test Method
Part A	**Description:**			
1	Sample was received in its characteristic form.			
Part B	**Analysis:**			
1	Taste	Natural Sweet	Natural Sweet	Organoleptic
2	• Mould<br>• Dirt & Scum<br>• Pieces of beeswax<br>• Fragments of bees and other insects<br>• Any other extraneous matter	Free from	Free from	Visual
3	Color, mm	Light Amber	Light to dark brown	Pfund color Grader
4	Specific Gravity at 27°C	1.41	1.35 (Min)	IS 4941
5	% Moisture	18.2	25 (Max)	IS 4941
6	% Total Reducing Sugar	76.96	65 (Min)	IS 4941
7	% Sucrose	0.87	5.0 (Max)	IS 4941
8	F/G Ratio	1.08	0.95 (Min)	IS 4941
9	% Total Ash	0.12	0.5 (Max)	IS 4941
10	% Acidity (As formic acid)	0.055	0.2 (Max)	IS 4941
11	HMF, mg/kg	71.78	80 (Max)	IS 4941
12	Fiehe's Test	Negative	Negative	IS 4941
13	**Drug Residue:**			
	Chloramphenicol, ppb	ND	0.3	LC-MS/MS
	Nitrofurans, ppb	ND	0.5	LC-MS/MS
	Sulphonamides, ppb	ND	5.0	LC-MS/MS
	Streptomycin, ppb	ND	5.0	LC-MS/MS
	Tetracycline, ppb	ND	5.0	LC-MS/MS
	Oxytetracycline, ppb	ND	5.0	LC-MS/MS
	Chlortetracycline, ppb	ND	5.0	LC-MS/MS
	Ampicillin, ppb	ND	5.0	LC-MS/MS
	Enrofloxacin, ppb	ND	5.0	LC-MS/MS
	Ciprofloxacin, ppb	ND	5.0	LC-MS/MS
	Erythromycin, ppb	ND	5.0	LC-MS/MS
	Tylosin, ppb	ND	5.0	LC-MS/MS

Remarks: The sample Confirms to FSSAI Specification as on above tested Parameters.

(Stamp: Kejriwal Bee Care India (P) Ltd., New Delhi 110048, Greater Kailash-II)

(Authorised Signatory)

Note:

1. The results listed refer only to the tested sample and applicable parameters.
2. This report shall not be reproduced except in full without written approval of KBC.

RO: W-42, GREATER KAILASH-II, NEW DELHI-110048, INDIA T: +91-11-29219677 / 78 / 79 F: +91-11-29210985 E: mail@kejriwalgroup.co.in
BO: EVEREST HOUSE-6E, 46C, JAWAHARLAL NEHRU ROAD, KOLKATA-700 071 T: +91-33-22884428/29 F: +91-33-22884429 E: kol@kejriwalgroup.co.in
FACTORY: VILLAGE JALALPUR, P.O. BANUR, RAJPURA, DISTT. PATIALA, ZIRAKPUR, PATIALA HIGHWAY, PUNJAB-140601, INDIA
TEL.: +91-1762-251411 | E-MAIL: kbc@kejriwalgroup.co.in
WEBSITE: www.kejriwalhoney.com, www.honeychew.com | CIN NO. UO1122DL2002PTC117220

Scanned with CamScanner
