# Supplementary material for: Effect of honey and insulin treatment on oxidative stress and nerve conduction in an experimental model of diabetic neuropathy Wistar rats
Source: PLoS One. 2021 Jan 15;16(1):e0245395. doi: 10.1371/journal.pone.0245395 (PMC7810291; doi:10.1371/journal.pone.0245395)
Supplement: Attachment — Submitted filename: Response to Reviewers.docx [file pone.0245395.s003.docx]

PONE-D-20-18538R1
Effect of honey and insulin treatment on oxidative stress and nerve conduction in an experimental model of diabetic neuropathy Wistar rats

Review Comments to the Author

Reviewer #1: In their revised version of their MS, Authors correctly addressed most of points I raised. Still, I do have several questions:

**Concerning point #11.** Lane 176 Authors wrote “Total antioxidant status (TAS) was measured by ferrous reducing antioxidant power [FRAP] assay”. I am not sure FRAP is sufficient to evaluate TAS. In your answer you submit reports that state FRAP assay is a test to assess total antioxidant power. Actually my point is FRAP, despite being a very good test to assess antioxidant capacity of a solution/molecule, is not sufficient to evaluate TAS. Additional tests should be used to be sure to evaluate TAS such as ORAC, DDPH…
**Answer:** We agree with the inputs of the learned reviewer that Oxygen Radical Absorbance Capacity (ORAC) and Diphenyl-β-picrylhydrazyl (DPPH) are better tests for estimating total antioxidant capacity. ORAC is the most widely recognized method of all the antioxidant assays, the reaction is simple in concept but it is more complex in practice. As there are some practical issues in performing ORAC such as careful control and monitoring of ORAC reaction temperature and careful control of oxygen are critical, we could not use this test in our study for estimating TAS.

In DPPH, the reaction needs longer duration. The method is based on the spectrophotometric measurement of DPPH concentration changes resulting from the DPPH reaction with an antioxidant. The amount of remaining DPPH in the examined system is a measure of the antioxidant activity of compounds. The study shows that the type and amount of solvent used for the antioxidant dissolution, water content, and hydrogen or metal ion concentration in the measuring system significantly influences the differences in the amount of unreacted DPPH. Due to this presented relationships, standardising the DPPH method shows the complexity of the problem, even in very simple DPPH/antioxidant systems, which is one of the reason for not selecting DPPH for measuring TAS.

However, reports suggests that FRAP assay is comparable with both of these methods (1,2) and used in various studies for measuring the TAS. The FRAP assay is simple and also feasible for us to do in our laboratory setup. Reagents of FRAP are inexpensive, of low toxicity and high stability. The procedure of FRAP is technically straightforward and the results are available within a few minutes of reagent/sample mixing (4min at the recommended reaction temperature to 37°C). Also, FRAP assay has high sensitivity and precision. Taking these points into consideration, we selected FRAP assay in our study for the estimation of TAS.

References:

1. Rao PVLNS, V.S.Kiranmayi, P.Swathi, Jeyseelan L, M.M.Suchitra, Bitla AR. Comparison of Two Analytical Methods Used For the Measurement of Total Antioxidant Status. JAA. 2015 Jun 6;1(1):22.
2. Dudonné S, Vitrac X, Coutière P, Woillez M, Mérillon J-M. Comparative Study of Antioxidant Properties and Total Phenolic Content of 30 Plant Extracts of Industrial Interest Using DPPH, ABTS, FRAP, SOD, and ORAC Assays. J Agric Food Chem. 2009 Mar 11;57(5):1768–74.

**Concerning point #21.** Authors should provide a table with information about regimen in term of composition for standard rodent chow (SRC) and SRC plus honey.
Authors give data in their answer but with no unit. The table with regimen composition and correct units should be included in the revised version of the MS.
There are still typos in the MS, Authors should proofread carefully before resubmitting.

**Answer:** SRC composition (with units) is included in the revised version of the MS

**Lane 420 authors wrote “hypolipedemic effect was moderate”**

**Answer:** Corrected

Reviewer #2: The authors have addressed fully comments from reviewer 1 and 2 but not entirely those from reviewer 3.

**Answer:** We have included all the corrections/suggestions given by the esteemed reviewers in the new-revised manuscript.

Reviewer #3: The reviewer and editor comments appear to have been addressed with care and I applaud your level of honesty in admitting when you simply cannot afford or accomplish specific methods. My concern is that beyond defending your manuscript to the editor and reviewers you made very little changes to the manuscript itself. I believe the comments are made to improve the quality of the manuscript and some of the motivations made need to be reflected in the published work. As such I believe the answers given satisfactory, but would like to see it reflected in the manuscript.

**Answer:** We are grateful to the suggestion of our revered reviewer. As per the suggestion of our esteemed reviewers we have included our responses to the previous review comments in the revised manuscript.
