# Supplementary material for: Effect of honey and insulin treatment on oxidative stress and nerve conduction in an experimental model of diabetic neuropathy Wistar rats
Source: PLoS One. 2021 Jan 15;16(1):e0245395. doi: 10.1371/journal.pone.0245395 (PMC7810291; doi:10.1371/journal.pone.0245395)
Supplement: Attachment — Submitted filename: Response to Reviewers.docx [file pone.0245395.s004.docx]

PONE-D-20-18538R2
Effect of honey and insulin treatment on oxidative stress and nerve conduction in an experimental model of diabetic neuropathy Wistar rats


6. Review Comments to the Author

**Reviewer #1:** In their revised version of the MS, Authors correctly addressed points I previously raised. Just few minor points:

In table 2 please use only 3 significant numbers.

- Same for table 3 and 4.

**Answer:** As per the esteemed reviewer’s suggestion, in table 2, 3 and 4 following decimal, only two significant numbers were given.

If this did not address the query of our esteemed reviewer, we request to provide one example table of the required change. As per the example we will make the changes and submit it as required.

**Reviewer #3:** Thank you for addressing the review comments. The only shortcoming that I can still point out is the need to show the dietary composition of SRC and Honey. This may be technically difficult, as the honey was dissolved in water. I however, feel it would be a improvement on the current version.

**Answer:** We thank our learned reviewer for his valuable comments and suggestion. The dietary composition of honey was not specifically estimated by us in the study as that was not our specific objective of the study. However, we asked the company to provide honey analysis report when they provided honey. We are herewith attaching the analysis of honey (FSSAI approved) as supplementary data for your review.
